# Online resources for improving the care and use of non-human primates in research

**DOI:** 10.5194/pb-3-33-2016

**Published:** 2016-07-26

**Authors:** Mark J. Prescott

**Affiliations:** National Centre for the Replacement, Refinement and Reduction of Animals in Research (NC3Rs), Gibbs Building, 215 Euston Road, London, NW1 2BE, UK

## Abstract

Published literature and scientific events provide opportunities to expand knowledge and develop skills in the care and use of non-human primates (NHPs) in research. Increasingly, these traditional routes of information exchange are being complemented by dedicated online resources aimed at sharing best practice in NHP care and use, and enhancing the training and professional development of laboratory staff working with NHPs. This article outlines some key online resources from the UK’s National Centre for the Replacement, Refinement and Reduction of Animals in Research (NC3Rs) and other organisations with an interest in NHP research, and the ways in which the resources can be integrated into staff training and research practices to enhance animal welfare, quality of science and application of the 3Rs.

## 1 Introduction

The Internet and widespread access to the World Wide Web has revolutionised the speed, ease and flexibility with which scientists and other laboratory professionals can share information, data and ideas. These developments, coupled with changes in academic publishing such as open-access journals and greater self-archiving of research outputs, and a movement for greater openness in animal research, have led to an increase in the amount of information available online about the use and care of non-human primates (NHPs) in the laboratory. This knowledge base provides an important opportunity for staff to improve the welfare of the animals in their care and the quality of science derived from them, as well as a means to enhance public support for NHP research. However, as with other forms of knowledge exchange, there is a need to consider the quality and timeliness of the information being shared.

The NC3Rs^1^ is committed to supporting the scientific community to implement the 3Rs (replacement, reduction and refinement of animal use). We have an extensive programme designed to support scientists, veterinarians and animal care staff who work with NHPs. As part of this programme, we have developed a series of freely accessible, high-quality online resources (Table 1) to enhance sharing of good practice in the husbandry and care of macaques and common marmosets, to improve understanding of their behaviour and welfare needs, and to optimise their use so that high-quality data are delivered from as few animals as possible. The online format of these resources has advantages over print publication, in that the content can be highly visual and rapidly updated, and in some cases there is functionality for feedback and discussion. This article describes these and other online resources and the ways in which they can be used to support application of the 3Rs and high standards in NHP care and use programmes.

## 2 Resources from the NC3Rs

### 2.1 The Macaque Website (www.nc3rs.org.uk/macaques)

The Macaque Website, launched in May 2015, covers the life history of the rhesus macaque *(Macaca mulatta)* and cynomolgus macaque *(Macaca fascicularis)*, their behaviour (such as postures, expressions, vocalisations), captive management (such as housing, enrichment, feeding, training) and tools for welfare assessment (behavioural, physiological and health indicators). It features over 200 images and videos, along with practical guidance, which is peer reviewed and referenced. Developed by the NC3Rs (Mark Prescott), with assistance from Emily Bethell and Caralyn Kemp of Liverpool John Moores University, the aim of this highly visual resource is not to provide a manual on macaque care and use, but instead to inspire, inform and share good practice to improve the welfare of these animals in laboratories worldwide. Individuals from 25 organisations internationally contributed material for the website, including major academic and industry laboratories and research field sites.

A good understanding of the behaviour of the species staff members work with is fundamental to fulfilling the refinement principle of the 3Rs. It underpins, for example, effective environmental enrichment strategies, empathetic human–animal interactions, and the ability to use behaviour to identify animals with compromised welfare before issues become serious. Many scientists, veterinarians and animal care persons working with macaques in research and testing do not have a formal training in animal behaviour, and initial mandatory training programmes may concentrate on occupational health and safety and conducting scientific procedures, at the expense of behavioural aspects. This can compromise the ability of research organisations to operate to best practice in their use and care of NHPs.

The comprehensive behaviour section of the Macaque Website provides a guide to social and non-social behaviours likely to be performed by captive macaques. It enables laboratory staff to better recognise, interpret and respond appropriately to macaque communication signals, which will provide a good basis for refining many aspects of their care and use. The welfare assessment section of the site also features a 1 h “Listen Again” presentation on recording behaviour, which provides the essential training required for more formal assessment of animal behaviour, such as that required in a small research project to evaluate the effectiveness of a new enrichment device. The presentation is also useful to students of primatology and animal behaviour. An interactive quiz to test knowledge of macaque behaviour has recently been added to the site.

Not everyone working with macaques in research has the opportunity to travel to other laboratories and to compare and contrast their infrastructure and practices with those of others – to see whether they are ahead or behind the curve with their animal management and to gather new ideas. The Macaque Website illustrates some exemplary accommodation and enrichment ideas instantly in photo and video format. In doing so, it helps to dispel some common myths – for example, that macaques used in experiments must be housed in small, squeeze-back cages, else they will be difficult to capture; that animals with head implants cannot be housed socially in enriched enclosures; and that destructible materials such as wood and cardboard are not safe as enrichment items. It demonstrates the high standards that can be achieved with sufficient awareness, will and resources and enables laboratories to benchmark their standards against those of other laboratories. To assist research establishments to implement what is shown on the site, there is practical advice – for example, recipes for cheap enrichment items, as well as ideas for incremental improvements in caging design where resources are limited. Such information is particularly useful for those working in NHP laboratories in developing countries.

The field of animal welfare science is constantly evolving and new, improved methods for assessing welfare emerge. The website pulls together some of the tools that can be used to identify individuals at risk of poor welfare and to evaluate the impact of refinements in research and husbandry procedures – from body condition and alopecia scoring systems, to haemodynamic, respiratory and blood clinical chemistry reference values. The NC3Rs is a major funder of new methods for assessing pain and distress in macaques, as well as new research materials that enable scientific procedures to be performed with reduced harm caused to the animals. As these research projects deliver practical tools for welfare assessment, they will be added to the website so that the latest techniques can be employed by others.

### 2.2 Common Marmoset Care (www.marmosetcare.com)

The Macaque Website is complemented by an earlier website on the behaviour and care of common marmosets *(Callithrix jacchus)*, called Common Marmoset Care. This site was developed by Claire Watson and Hannah Buchanan-Smith at the University of Stirling, with funding and input from the NC3Rs (Mark Prescott) and Primate Society of Great Britain. Launched in October 2011, the interactive site provides a wealth of information on how best to care for these small New World monkeys in captivity. The key audiences are researchers, private owners, educators, and zoo and laboratory professionals. There is a strong presumption on the website against the keeping of these animals as pets.

Like the Macaque Website, Common Marmoset Care is highly visual, with over 100 images and videos. The site includes a comprehensive section on understanding marmoset behaviour, with information to help users of the website to interpret the actions, facial expressions, body postures and vocalisations of these animals, as well as an interactive quiz to test acquisition of knowledge. The section “In the Wild” contains a gallery of images of common marmosets in their natural habitat, and a 5 min video illustrating a day in the life of wild marmosets in Brazil.

There is also extensive information on aspects of marmoset husbandry and use in research, such as housing, grouping, feeding, handling and interactions with humans. One of the most valuable and unique features of the website is the large collection of videos demonstrating practical environmental enrichment strategies for these animals – from objects to encourage play and natural foraging behaviour, to ways to stimulate the senses and encourage exploration.

As with the Macaque Website, the marmoset site can be used to gather enrichment ideas, benchmark housing standards, assess NHP welfare using behavioural indicators and contribute to staff training. Whilst not as well referenced as the Macaque Website, there is a list of links to useful publications. Additional video material on the handling of common marmosets and their use in Parkinson’s disease research is under development.

### 2.3 Chronic Implants Wiki (www.ciwiki.net)

The Chronic Implants Wiki (CIwiki) represents a novel approach to sharing of information within the NHP neuroscience community. The focus is on refinement of the implanted devices used to enable electrophysiological recordings from the brain in long-term neuroscience studies, such as head posts and recording chambers. The NC3Rs has played an important role in encouraging innovation in this area, through workshops, research funding and prizes. Like the well-known Wikipedia, the CIwiki relies upon contributions from registered users, who can continuously edit the pages within the wiki, but unlike Wikipedia, its pages are visible only to authenticated individuals. To register to use the wiki, complete the short registration form on the home page and email it to the moderator.

Recent years have seen considerable innovation in implant design, utilising advances in medical technology and engineering to produce devices which are more tissue friendly, integrating better with the skull, with reduced risk of infection at the implant margin. Such designs benefit not only animal welfare but also science, since infection and inflammation can lead to breakage or loosening of the device and animals having to be temporarily or permanently removed from the study. Whilst improvements in methodology are sometimes reported in the peer-reviewed literature, this is not always the case, perhaps because they carry less prestige than those reporting new scientific discoveries. The wiki was the idea of Daniel Adams, University of California, San Francisco, and is managed by the NC3Rs (Mark Prescott). It provides a secure forum for sharing of refinements to established techniques, no matter how incremental, rapidly and easily, with the prospect of feedback. Information on technical complications and flawed approaches is also useful to de-risk future ventures and avoid repetitions of failures.

The wiki was launched in December 2015 and currently contains practical information (e.g. images, technical notes, journal articles) on issues such as implant design, construction materials, surface coatings, holding mechanisms and infection control, as well as information on some non-invasive approaches for head immobilisation. The content and utility of the wiki will expand as there is increasing active engagement from the international neuroscience community.

### 2.4 Welfare of Non-Human Primates hub (www.nc3rs.org.uk/welfare-non-human-primates)

A hub of the main NC3Rs website includes additional information on opportunities to reduce harms and improve the welfare of NHPs used in research, as identified by various scientific working groups of the NC3Rs. For example, there are recommendations to refine the use of food and fluid control as motivation tools for macaques used in behavioural neuroscience, to incorporate positive reinforcement training techniques into husbandry and scientific procedures in order to reduce distress, to wean macaques at an appropriate age for behavioural competence, and to safely rehome exlaboratory primates where this is in the best interests of the individuals involved. Links are provided to the open-access, peer-reviewed journal articles, which contain the background information and evidence bases to support the recommendations for refinement. Also in this area are details of the annual NC3Rs event dedicated to promoting ways to improve primate welfare (Primate Welfare Meeting), and the NC3Rs guidelines “Primate accommodation, care and use”, which set out principles of good practice in the care and use of NHPs. Implementation of the principles in the guidelines is a condition of receiving funds from any of the major UK public funding bodies of NHP research.

### 2.5 Animals in Drug Discovery and Development hub (www.nc3rs.org.uk/animals-drug-discovery-and-development)

The NC3Rs (principally Kathryn Chapman) has also led a number of projects to challenge the need for NHP studies in drug discovery and development, and to optimise study designs where their use is necessary for the testing of new pharmaceuticals and biologics. This has involved pre-competitive data sharing between more than 30 industry companies, contract research organisations and regulators, especially in Europe and the USA. In some cases, the evidence base generated has led to regulatory change in addition to changes in policy and practice within the participating organisations. The peer-reviewed output from these projects is conveniently collated in a hub of the main NC3Rs website. These include new opportunities for reducing the use of NHPs in the development of biotherapeutics, as recovery animals on toxicity studies, for the study of drug abuse potential, and for the prediction of pharmacokinetics for compounds cleared by hepatic cytochrome P450 enzymes. There is also published information on reducing the upper limit for body weight loss in maximum tolerated dose studies, and allowing social housing during telemetry recordings in safety pharmacology and toxicology studies.

### 2.6 NACWO Network email discussion group (email: mark.prescott@nc3rs.org.uk)

The UK Animals (Scientific Procedures) Act 1986, amended 2012, requires that establishments breeding and/or using animals in scientific procedures appoint a Named Animal Care and Welfare Officer (NACWO), who has responsibility for the day-to-day care of the animals (or specific animal species within the facility). In 2013, the NC3Rs established a network for NACWOs working with NHPs to enable sharing advice, experience and ideas, via an annual meeting, exchange visits and a dedicated email discussion group. Recently a decision was made to expand the network to include people working in similar roles outside of the UK. Animal welfare officers working with NHPs can join the email discussion group by contacting mark.prescott@nc3rs.org.uk (give your full name, job position and work email address).

### 2.7 Experimental Design Assistant (https://eda.nc3rs.org.uk/)

Another NC3Rs resource worthy of note is the Experimental Design Assistant (EDA). Unfortunately, evidence exists to show that many animal studies, including those utilising NHPs, are poorly designed, analysed and reported and that this has significant implications in terms of reproducibility and the translation of findings into clinical benefits (Kilkenny et al., 2009). The EDA was developed to address these problems and guide researchers through the design of their experiments, helping to ensure that they use the minimum number of animals consistent with their scientific objectives, incorporate measures to reduce subjective bias, and choose appropriate statistical analysis methods. It complements the NC3Rs’ ARRIVE guidelines (Kilkenny et al., 2010; www.nc3rs.org.uk/ARRIVE), which were developed to improve the reporting of in vivo studies, in order to maximise the information published and minimise unnecessary studies. To date, the ARRIVE guidelines have been endorsed by more than 1000 journals internationally and many research funders and learned societies.

Launched in 2016, the EDA is an online web application, with an associated website which contains a wealth of information and advice on experimental design. It was developed by the NC3Rs (Nathalie Percie du Sert) in collaboration with in vivo scientists and statisticians from academia and industry, along with software developers specialising in artificial intelligence. Having created a user account, researchers from a wide variety of disciplines can use the application to build a visual representation of their intended experiment (the EDA diagram), based on a pre-established ontology. The EDA diagram is a more comprehensive and transparent means of summarising an experiment than a conventional text description. A “critique” function enables users to receive feedback and advice on their diagram (based on an extensive dataset of rules), in order to optimise their experimental design and analysis. The feedback may include, for example, highlighting the implications of some of the design choices made, or pointing out issues with internal consistency such as where two variables are confounded. Built into the system is dedicated support for randomisation, blinding and sample size calculation. The final, improved and transparent experimental plan can then be shared directly with colleagues (e.g. collaborators, students, supervisors) from within the EDA.

## 3 Resources from other organisations

Primatological and primate veterinary societies also have a presence online (see Table 2). In additional to information on upcoming scientific meetings, research funding opportunities and prizes, some society websites include guidelines and other unique content. For instance, the International Primatological Society Captive Care Committee has published *International Guidelines for the Acquisition, Care and Breeding on NonHuman Primates* (McCann et al., 2007), which have been translated into French, Spanish, Chinese and Japanese (http://www.internationalprimatologicalsociety.org/publications.cfm).

The Association of Primate Veterinarians produces education resources for its members, some of which are free to download – for instance, a series of guidelines on topics relating to the use of NHPs in biomedical research from its Scientific Advisory Committee, and a “NHP Formulary” of drug doses and normal physiological values for commonly used species (https://www.primatevets.org/education).

Some learned societies have LinkedIn Groups, which are useful for building professional networks and for keeping up to date on NHP-related news (see Table 2). One advantage of social media channels such as LinkedIn is that announcements are actively “pushed” to the members, avoiding busy people having to browse websites for new content. Hence the NC3Rs regularly posts about its NHP activities (e.g. upcoming events, new research papers) to LinkedIn Groups like NHP Toxicology, Society for Neuroscience, Animal Models in Neurosciences, Association of Primate Veterinarians and European Primate Veterinarians. All of these groups provide the opportunity to pose questions to the entire membership, encouraging discussion and debate, or to email individual members privately.

Many primatological societies publish their own journals, which are a rich source of information on NHP behaviour and care in captivity. Some key journals are listed in Table 2; a more extensive list of links to primatological journals and newsletters can be found on the Primate-InfoNet website (http://pin.primate.wisc.edu/news/journals/journals.php). However, much information relevant to the care and management of NHP species used in biomedical research, and on applying the 3Rs to this use, is published in general biology, laboratory animal science and animal welfare journals, as well as discipline-specific journals, such as those within neuroscience, immunology and infectious disease, psychology and toxicology. Therefore, it is important to conduct a well-conceived literature search of mainstream bibliographic databases and academic search engines, such as PubMed and Scopus, before embarking on new research projects and reviews of facility practices so that all of the latest relevant information can be taken into account. Guidance on searching for alternatives (all 3Rs) is provided by Altweb (http://altweb.jhsph.edu/resources/searchalt/) and the Animal Welfare Information Centre (https://awic.nal.usda.gov/awic-tips-searching-alternatives-animal-research-and-testing-0).

Increasingly, research papers are freely available in online repositories, as scientists publish their work in open-access journals in order to comply with the mandates of funding bodies and to give the work greater visibility and impact. Where papers are not available as open access or via an institutional subscription, they can sometimes be obtained from the author’s ResearchGate profile (https://www.researchgate.net/). It is also worth searching academic search engines too, as there are many good books available for free online, such as *The Psychological Wellbeing of Nonhuman Primates* (National Research Council, 1998).

Some regulatory and funding agencies have created online resources on aspects of NHP care and use – mainly bibliographic databases – either directly or supported via grant funding. Key resources are listed in Table 2. These provide a gateway to relevant literature (including national legal standards and guidance documents) and in some cases to opportunities for training and collaboration.

## 4 Concluding remarks

Research with NHPs is a complex undertaking requiring a team approach from people with varied and specialist knowledge and skills. Conducting the very best science depends on keeping abreast of the latest knowledge and techniques, not just in research methods and scientific procedures, but also in NHP behaviour, management and welfare, as increasingly studies demonstrate a link between good animal welfare and quality of science (Graham et al., 2011; Guth et al., 2009; Reinhardt, 2004; Schnell and Gerber, 1997; Shirasaki et al., 2013). Researchers, veterinarians and animal care staff should therefore supplement their initial training with continuing learning and professional development, part of which can be provided by resources online.

To maximise use of these resources and the information they provide, they should be integrated into local research and animal care practices. This can be done by, for example, incorporating them into the content and delivery of staff and student training courses, linking to them from relevant areas of the organisation’s intranet, raising awareness with e-newsletters and/or posters, and using the material to review and revise standard operating procedures. Ultimately, this will help to raise standards of NHP care and use, facilitate research collaborations, and address societal concerns about NHP research.

## Figures and Tables

**Table 1:** Summary of NC3Rs online resources relevant to the care and use of NHPs.

Resource	Type	Description
The Macaque Website www.nc3rs.org.uk/macaques	Website	Information-rich, highly visual and referenced website on the behaviour, captive management and welfare assessment of macaques.
Common Marmoset Care www.marmosetcare.com	Website	Information-rich and highly visual website on the behaviour, natural history and captive care of common marmosets.
Chronic Implants Wiki www.ciwiki.net	Wiki	Wiki for exchange of technical information aimed at refining the use of chronic implants used in long-term neuroscience studies.
Welfare of Non-Human Primates www.nc3rs.org.uk/ welfare-non-human-primates	Website hub	Hub of the main NC3Rs website showcasing the activities and outputs from the centre’s office-led programme on improving NHP welfare.
Animals in Drug Discovery and Development www.nc3rs.org.uk/animals-drug-discovery-and-development	Website hub	Hub of the main NC3Rs website collating new opportunities to reduce and refine use of animals (including NHPs) in the pharmaceutical industry, generated from NC3Rs-industry collaborations.
NACWO Network Email: mark.prescott@nc3rs.org.uk	Email discussion group	Email discussion group for animal welfare officers (in the UK, Named Animal Care and Welfare Officers – NACWOs) working with NHPs in the UK and elsewhere.
Experimental Design Assistant (EDA) https://eda.nc3rs.org.uk/	Website and app	Web application and supporting website to assist researchers in the planning of experiments, ensuring robust study design and reproducible findings.

**Table 2:** Summary of some other online resources relevant to the use and care of NHPs.

Resource	Description
Learned societies and networks	
International Primatological Society (IPS) http://www.internationalprimatologicalsociety.org/	Society created to encourage NHP research, facilitate cooperation among scientists, and to promote the conservation of NHP species.
American Society of Primatologists (ASP) https://www.asp.org/	Society of primatologists and others involved in the discovery and exchange of information regarding NHPs.
European Federation for Primatology (EFP) http://www-3.unipv.it/webbio/efp/efp.htm	Umbrella body of national primatological societies within Europe.
Primate Society of Great Britain (PSGB) http://www.psgb.org/	The UK’s national primatological society, dedicated to the advancement of primate research, conservation and captive care.
Association of Primate Veterinarians (APV) https://www.primatevets.org/ https://www.linkedin.com/groups/4700626	Network of veterinarians advancing and promoting the science, medicine, management and humane care of NHPs.
European Primate Veterinarians (EPV) http://www.euprimvets.eu/ https://www.linkedin.com/groups/4703506	Network of veterinarians working with NHPs in European primate centres in academia, industry and other sectors.
NHP Toxicology https://www.linkedin.com/groups/4669050	LinkedIn group established to share diagnostic techniques and methods used in regulatory-driven preclinical safety assessment studies using NHPs.
Marmoset Research Group of the Americas (MaRGA) http://www.primate.wisc.edu/MaRGA/	Network of primatologists, biomedical researchers and veterinarians with an interest in marmosets and tamarins.
Primatology journals	
*American Journal of Primatology* http://onlinelibrary.wiley.com/journal/10.1002/(ISSN)1098-2345	Official journal of the American Society of Primatologists.
*Folia Primatologica* http://content.karger.com/Journal/Home/223842	Official journal of the European Federation for Primatology and the Primate Society of Great Britain.
*International Journal of Primatology* http://link.springer.com/journal/10764	Official journal of the International Primatological Society.
*Journal of Medical Primatology* http://onlinelibrary.wiley.com/journal/10.1111/(ISSN)1600-0684	Online-only journal concerning NHPs as models to study, prevent, and/or treat human diseases.
*Laboratory Primate Newsletter* (open access) http://www.brown.edu/Research/Primate/	Archive of short articles with practical information on running an NHP facility, published between 1962 and 2011.
*Primate Biology* (open access) http://www.primate-biology.net/	Open-access journal for research in all scientific areas involving NHPs.
*Primate Report* (open access) http://www.dpz.eu/en/unit/library/downloads/primate-report.html	Journal of the German Primate Center (DPZ). Archive of papers and abstracts published between 2002 and 2009.
*Primates* http://link.springer.com/journal/10329	Official journal of the Japan Monkey Centre, published in cooperation with the Primate Society of Japan.
Dedicated resources	
Primate Info Net http://pin.primate.wisc.edu/	Comprehensive website of NHP related information, maintained by the Wisconsin Primate Research Center (WPRC) Library at the University of Wisconsin-Madison. Includes news, meetings, fact sheets, job opportunities, audio visual resources, and a list of links on handling, care and enrichment: http://pin.primate.wisc.edu/research/vet/
International Directory of Primatology http://pin.primate.wisc.edu/idp/	Directory of people, organisations, businesses and field sites active in NHP research, education and conservation. Part of the Primate Info Net.
PrimateLit http://primatelit.library.wisc.edu/	Searchable database of primatological literature published between 1940 and 2010. Part of Primate Info Net and originally supported by an NIH grant.
Environmental Enrichment for Nonhuman Primates Resource Guide (updated July 2015) https://awic.nal.usda.gov/environmental-enrichment-nonhuman-primates-resource-guide	A bibliographic resource on NHP enrichment, including article links, organisations, and product information. Maintained by the Animal Welfare Information Center.
Annotated Database on Environmental Enrichment and Refinement of Husbandry for Nonhuman Primates (updated April 2016) http://awionline.org/content/primate-enrichment-database	Searchable database of published articles, books and other resources on the housing, enrichment and handling of NHPs. Maintained by the Animal Welfare Institute.
The Shape of Enrichment, Inc. http://www.enrichment.org/	Non-profit corporation set up to further animal enrichment efforts worldwide. Produces a quarterly publication, The Shape of Enrichment, dedicated to sharing enrichment ideas among animal caretakers, and organises conferences and workshops.
EUPRIM-Net website http://www.euprim-net.eu/	European Commission-funded initiative, aimed at linking the infrastructure and expertise of European primate centres in order to advance the 3Rs. The website includes information on research activities, training courses and biobanks.
NPRC Research and Capabilities Inventory website http://nprcresearch.org/primate/	Website aimed at facilitating research collaborations with the seven United States national primate research centres.

## Footnotes

1 The NC3Rs is an independent scientific organisation established by government in the United Kingdom to discover, develop and promote new ways to replace, reduce and refine the use of animals in science (the 3Rs). It is the largest funder of 3Rs research in the UK and also works in collaboration with scientists and organisations from across the life sciences sector, nationally and internationally, to generate an evidence base for changes in policy, regulations and practice which advance the 3Rs and support better science.
